# Comparative genome analysis of endophytic *Bacillus amyloliquefaciens* MR4: a potential biocontrol agent isolated from wild medicinal plant root tissue

**DOI:** 10.1007/s13353-024-00905-9

**Published:** 2024-09-30

**Authors:** Kaiying Yang, Xianxing Dai, Zulihumar Maitikadir, Huijiang Zhang, Haiting Hao, Chengcai Yan

**Affiliations:** 1 Scientific Observing and Experimental Station of Crop Pests in Alar, Ministry of Agriculture/Key Laboratory of Integrated Pest Management (IPM) of Xinjiang Production and Construction Corps in Southern Xinjiang, College of Agronomy, Tarim University, Alar, 843300 Xinjiang China; 2 Key Laboratory of Genetic Improvement and Efficient Production for Specialty Crops in Arid Southern Xinjiang of Xinjiang Corps, College of Agronomy, Tarim University, Alar, 843300 Xinjiang China

**Keywords:** *Bacillus amyloliquefaciens*, Biological control; Whole-genome sequencing; Endophyte; Comparative genomics

## Abstract

In this investigation, 396 endophytic bacterial strains from six indigenous medicinal plant species within the Xinjiang Tumor Peak National Nature Reserve were subjected to screening. The strain MR4 emerged as a noteworthy contender, demonstrating pronounced biocontrol capabilities coupled with exceptional cold tolerance. Through morphological scrutiny and comprehensive genomic sequencing, MR4 was identified as *Bacillus amyloliquefaciens*. Antagonistic assays revealed MR4’s efficacy in suppressing the causative agents of cotton wilt and verticillium wilt, achieving inhibition rates surpassing 50%. Analyses, underpinned by PCR methodologies, indicated MR4’s capacity to biosynthesize a minimum of eight distinct antimicrobial agents. The whole-genome sequencing data indicated that *B. amyloliquefaciens* MR4 had the genome size and GC content of 4,017,872 bp and 47.14%, respectively, and 4191 coding genes were identified. The genome consists of a single chromosome and one plasmid. Moreover, it was augmented by annotations from various databases, including GO, KEGG, and COG. The pathogenicity of MR4 undergoes evaluation, while predictions concerning MR4’s secondary metabolites have disclosed gene clusters for 13 varieties of these compounds, with particular emphasis on surfactins and fengycin. Comparative analyses with four paradigmatic strains shed light on MR4’s genomic composition and its phylogenetic lineage within the *Bacillus* genus. The genomic data pertaining to MR4 have been duly submitted to the NCBI GenBank, bearing the accession numbers CP146236 (Chr1) and CP146237 (plas1). This study endeavors to furnish potent microbial resources for the biocontrol and enhancement of plant growth, thereby providing a theoretical groundwork for MR4’s agronomic utilization.

**Graphical Abstract:**

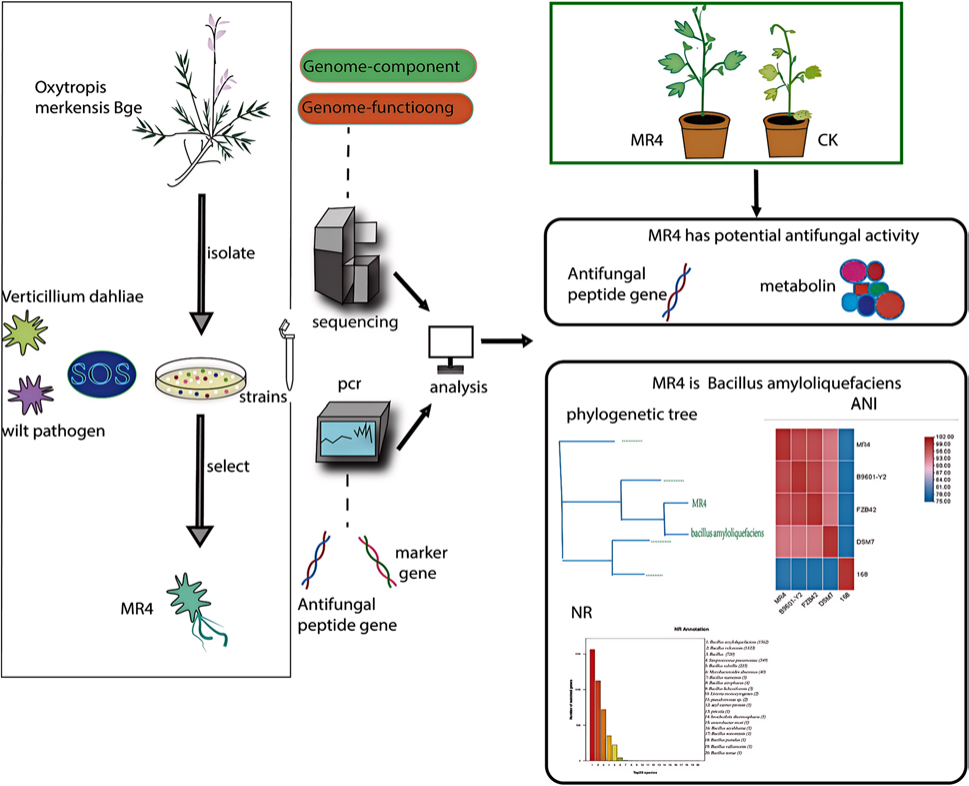

**Supplementary Information:**

The online version contains supplementary material available at 10.1007/s13353-024-00905-9.

## Introduction

Plant diseases significantly hinder agricultural yield, stability, and quality, with traditional chemical pesticides posing risks like resistance, environmental pollution, and health issues (Shi 2004). Consequently, there is a pressing demand for benign, effective alternatives to these chemical measures. Biocontrol, particularly through *Bacillus amyloliquefaciens*, emerges as a promising solution, demonstrating remarkable disease management in crops such as rice, tomatoes, and cucumbers. Research on 31 endophytic strains from chili peppers has shown their antimicrobial principles like cucumber and banana wilt (He et al. 2002). Our recent findings reveal that the MR4 strain exhibits a 72.02% antagonism rate against cotton verticillium wilt and 58.8% against *Fusarium oxysporum*, highlighting its biocontrol potential.

*Bacillus amyloliquefaciens* (*B. amyloliquefaciens*), a Gram-positive, spore-forming bacterium, is recognized for its starch-decomposing ability. Found in soil, water, and the rhizosphere, it is a valuable microbial resource for biocontrol, enzyme production, pesticide development, and environmental protection (Ngalimat et al. 2021). It produces antibiotics such as lipopeptides (iturin and surfactin) and polypeptides, offering broad-spectrum antifungal activity against fungi, bacteria, and some viruses (Ji et al. 2013). Beyond pathogen inhibition, it boosts plant defense mechanisms and competes with pathogens in the rhizosphere, thereby enhancing plant growth and disease resistance (Borriss et al. 2011). However, the majority of *B. amyloliquefaciens* isolates are obtained from soil. While it plays a crucial role in enhancing soil nutrient effectiveness and altering soil microbial communities, the complexity of the environment increases competition for resources and the risk of contamination, resulting in low isolation purity, difficulty in ensuring strain diversity and stability, significant environmental impacts, and substantial resource investment, potentially disrupting soil ecological balance.

Endophytes, compared to exogenous microbes, exhibit significant advantages due to their stable symbiotic relationships and mutual adaptability with host organisms, enabling them to play vital roles within host organisms. This functional synergy allows endophytes to provide essential functions such as nutrition provision and immune system regulation, while also playing a crucial role in protecting hosts from external pathogen invasion. The symbiotic relationship between endophytes and hosts promotes mutual evolution, forming a more complex and intimate interaction pattern, providing lasting advantages for host growth, health, and survival. Research indicates that utilizing endophytes isolated from medicinal plants and vincristine alkaloids establishes a biotransformation model, resulting in the isolation of endophytic bacteria capable of producing various secondary metabolites with pharmacological effects such as anti-inflammatory properties. Furthermore, a considerable number of strains with high antibacterial activity have been isolated from endophytic fungi obtained from Aconitum plants. Medicinal plant endophytic fungi, as a major source of endophytic fungi with antibacterial activity in medicinal plants, play a significant role in controlling the growth of one or more pathogens, which is essential for the prevention and treatment of plant diseases.

In the Tomur Peak National Nature Reserve of Xinjiang, China, our screening of 396 endophytic bacteria from six wild medicinal plants led to the discovery of MR4. This strain, noted for its biocontrol capabilities and cold resistance, undergoes rigorous evaluation through morphology, whole-genome sequencing, and PCR-based antifungal gene detection. Comprehensive genome analysis, including annotations from GO, KEGG, and COG databases, alongside investigations into its antagonistic potential and secondary metabolites, aims to unlock MR4’s biocontrol mechanisms and understand its key genes. This endeavor not only addresses the shortcomings of chemical pesticides but also enriches the pool of sustainable agricultural practices.

## Materials and methods

### Isolation and screening of bacterial strains

In July 2023, rhizosphere samples from six medicinal plants were collected at Tumor Peak in Xinjiang, China, from an elevation of 2520 m (coordinates 80°23′3″E, 41°44′2″N). Using tissue separation techniques, endophytic bacteria were isolated and purified from plants (see Supplementary Material 1 for methods). The antagonistic effects of these bacteria against pathogens were assessed via the plate confrontation method. The assessment involved culturing activated bacterial strains in LB liquid medium at 30 °C with 200 rpm agitation for 16 h. A 5-mm pathogen block was placed at the center of a PDA plate, with 2.5 µl of bacterial strain inoculated symmetrically 25 mm away. Control plates were inoculated with only the pathogen. Plates were incubated inverted at 28 °C until the pathogen covered control plates, at which point the inhibition rate was calculated as follows: relative inhibition rate (%) = [(control radial growth − treated group radial growth)/control radial growth] × 100%.

### Construction of phylogenetic trees and nucleotide sequence alignment

To determine the taxonomic status of strain MR4, the 16S rRNA and housekeeping gene (gyrA) sequences of strain MR4 were submitted to the GenBank database (CP146236 (Chr1) and CP146237 (plas1)) for BLAST analysis (Borriss et al. 2011). The top ten strains with high sequence similarity were selected as references. Based on the maximum likelihood principle, phylogenetic trees were constructed for these sequences (Chen et al. 2018). Additionally, complete genome sequences of strains *B. amyloliquefaciens* FZB42, *B. amyloliquefaciens* DSM7, *B. amyloliquefaciens* B9601-Y2, and *B.* *subtilis* 168, whose genome sequencing data have been uploaded to NCBI, were downloaded. The basic sequence characteristics between MR4 and these strains were compared, and a heatmap based on average nucleotide identity (ANI) was generated (Arguelles-Arias et al., 2009).

### Antifungal activity measurement of *B. amyloliquefaciens* MR4 sterile fermentation broth and bacterial suspension against cotton *fusarium* and verticillium wilt pathogens

To further ascertain the biocontrol efficacy of MR4, its antifungal properties were reevaluated. The influence of MR4’s sterile fermentation broth on the growth of cotton fusarium wilt and verticillium wilt pathogen colonies was assessed using a co-cultivation approach. Moreover, the effect of an MR4 suspension on these pathogen colonies’ growth was examined via confrontation culture techniques (for detailed methods, refer to Supplementary Material 1).

### Detection of antifungal activity gene in strain MR4

Building upon prior research into genes associated with the biosynthesis of antimicrobial agents in the *Bacillus* genus, this study leveraged the complete genomic DNA of the MR4 strain as a framework to investigate its potential antifungal capabilities. Our investigation primarily centered on nine genes potentially harbored by the MR4 strain, which are responsible for the production of antimicrobial substances such as surfactin, fengycin, iturin, bacillomycin, mycosubtilin, bacilysin, bacillaene, difficidin, and bacillibactin (Lin and Liao 2013). To detect the gene sequences of these substances, we designed a series of specific primers (Table 1), employing polymerase chain reaction (PCR) technology (Zhou et al. 2022; Xu et al., 2018; Zhu et al. 2020; Wang et al. 2022).

**Table 1:** Primers for gene amplification related to antifungal substances in strain MR4

Lipopeptides	Gene	Primers	Sequences (5′–3′)	PCR product size (bp)
Surfactin	*srfAA* *srfAD*	srfAA-F srfAA-R srfAD-F srfAD-R	GCCCGTGAGCCGAATGGATAAG CCGTTTCAGGGACACAAGCTCCG CCGTTCGCAGGAGGCTATTCC CCGTTCGCAGGAGGCTATTCC	1600 1300
Fengycin	*fenB* *fenD*	fenB-F fenB-R fenD-F fenD-R	CTATAGTTTGTTGACGGCTC CAGCACTGGTTCTTGTCGCA TTTGGCAGCAGGAGAAGTTT GACAGTGCTGCCTGATGAAA	1600 1600
Iturin	*ituA* *ituC* *ituD*	ituA-F ituA-R ituC-F ituC-R ituD-F ituD-R	ATGTATACCAGTCAATTCC GATCCGAAGCTGACAATAG CCCCCTCGGTCAA GTGAATA TTGGTTAAGCCCTGATGCTC ATGAACAATCTTGCCTTTTTA TTATTTTAAAATCCGCAATT	1047 594 1203
Bacillomycin	*bmyA*	bmyA-F bmyA-R	AAAGCGGCTCAAGAAGCGAAACCC CGATTCAGCTCATCGACCAGGTAGGC	1200
Mycosubtilin	*mycB*	mycB-F mycB-R	ATGTCGGTGTTTAAAAATCAAGTAACG TTAGGACGCCAGCAGTTCTTCTATTGA	2000
Bacilysin	*bacAB* *bacD* *bacA*	bacAB-F bacAB-R bacD-F bacD-R bacA-F bacA-R	CAGCTCATGGGAATGCTTTT CTCGGTCCTGAAGGGACAAG CTTCTCCAAGGGGTGAACAG TGTAGGTTTCACCGGCTTTC GTGAAGGCCGTACTTTTGTCTGGC GGGGGGAAATACAGCTTCAGGGC	500 815 1200
Bacillaene	*baeS*	baeS-F baeS-R	CGCAAAAGCTCTTCGACCGCCGTC CTCTCGTGCCGTCGGAATATCCGC	1550
Difficidin	*dfnA*	dfnA-F dfnA-R	GGTGCGGCATGAAGATTTGAGATCACCG GGAGAGCACTTCAATTCCGACGTTGACC	1950
Bacillibactin	*dhbA*	dhbA-F dhbA-R	CGCCTAAAGTAGCGCCGCCATCAACGC CCGCGATGGAGCGGGATTATCCG	1350

### MR4 genome sequencing, assembly, and genome annotation prediction

The genomic DNA of the isolated endophytic bacteria was extracted via the SDS method and evaluated through agarose gel electrophoresis, with quantification conducted using the Qubit® 2.0 Fluorometer (Thermo Scientific) (Li et al. 2010). The MR4 genome was sequenced utilizing both the Nanopore PromethION platform and Illumina NovaSeq PE150, facilitated by Novogene Bioinformatics Technology Co., Ltd., Beijing (Li et al. 2008). Assembly of PE150 and Nanopore data was achieved with Unicycler software, followed by alignment to assembled sequences for sequencing depth distribution analysis. Sequences were classified as chromosomal or plasmid based on length and alignment, including checks for circular genomes. Initially, SPAdes software assembled the second-generation data into a scaffold, enhanced by third-generation data via miniasm and Racon, leveraging long-read data for bridging (Bankevich et al. 2012; Simpson et al. 2009). Unicycler resolved conflicts by selecting higher-quality bridges, ensuring cotig ends overlap and employing gene markers for default initiation site correction, resulting in seamless, complete sequences.

Gene prediction utilized GeneMarkS, with repetitive sequences identified by RepeatMasker and tandem repeats analyzed via Tandem Repeats Finder (Besemer et al. 2001). tRNA genes and rRNA genes were predicted using tRNAscan-SE and rRNAmmer, respectively, while snRNAs were identified through BLAST and Rfam database comparisons (Stanke et al. 2008; Lowe and Eddy 1997). Genomic islands and transposons were predicted using IslandPath-DIOMB and transposon PSI, respectively. Phage presence was assessed with PHAST, and CRISPR arrays were identified using CRISPRFinder (Hsiao et al. 2003; Grissaet al. 2007).

### Gene function annotation

We utilized seven databases for gene function prediction: GO (Gene Ontology), KEGG (Kyoto Encyclopedia of Genes and Genomes), COG (Clusters of Orthologous Groups), NR (Non-Redundant Protein Database), TCDB (Transporter Classification Database), and Swiss-Prot (Grissa et al. 2007; Kanehisa et al. 2006; Galperin et al. 2015). A genome-wide Blast search across these databases was conducted using criteria of an *E*-value less than 1e-5 and a minimum alignment length percentage over 40%. Secretory proteins were predicted using the Signal P database, and secretion systems types I–VII in pathogenic bacteria were predicted with EffectiveT3. Additionally, secondary metabolite gene clusters were analyzed with antiSMASH (Medema et al. 2011).

For antagonistic bacteria, we also performed analyses of pathogenicity and drug resistance, utilizing the PHI (Pathogen-Host Interactions) database, VFDB (Virulence Factor Database), and ARDB (Antibiotic Resistance Genes Database). Carbohydrate-active enzymes were predicted using the Carbohydrate-Active enZymes Database (Martin et al. 2015; Cantarel et al. 2009).

### Genomic components and collinearity analysis of MR4 and four strains of *Bacillus*

Complete genome sequences of strains *B. amyloliquefaciens* FZB42, *B. amyloliquefaciens* DSM7, *B. amyloliquefaciens* B9601-Y2, and *B. subtilis* 168, whose genome sequencing data have been uploaded to NCBI, were downloaded. The basic sequence characteristics between MR4 and these strains were compared. Using Mauve, the collinearity relationship of homologous genes between MR4 and these four model strains was constructed. This analysis enabled a comparison of genomic differences at the molecular level and facilitated an in-depth analysis of the genetic characteristics of MR4 (Rokas et al. 2018).

### Pot culture experiment design

Select a suitable number of uniform, plump seeds from cotton varieties Xin Hai 21 and Xin Lu Zhong 62. Soak the selected seeds in alcohol for 4–5 min, then remove them and rinse 2–3 times with sterile water. Wipe off the moisture with gauze. Lay 2–3 layers of gauze in a germination box, evenly distribute the treated seeds on top, cover with plastic wrap, and close the lid. Allow the seeds to germinate for 4–7 days. Once germinated, fill sterile pots with sterilized, sterile soil. Carefully transplant the germinated seeds into the pots, planting 3–4 seedlings per pot. Water the pots with an appropriate amount of sterile water and place them in a well-lit environment for cultivation. Water regularly, keeping the soil moist but not overly wet.

The strain MR4 will be inoculated onto solid LB agar and incubated in a 30 °C incubator for 24 h. Afterward, transfer the cultured MR4 strain to LB liquid medium and incubate on a shaker at 180 rpm at 30 °C for 16–20 h to prepare the MR4 bacterial suspension. When the cotton seedlings reach a height of 3–4 cm, dilute the prepared MR4 bacterial suspension with sterile water to a concentration of 10 − 7 for root irrigation treatment. Set up three replicates for each treatment in the experimental group, with an additional three pots serving as untreated blank controls.

After 15–20 days of cultivation, measure and record the following growth indicators of the cotton plants: number of leaves, root coefficient, aboveground length, underground length, total length, fresh weight, aboveground fresh weight, underground fresh weight, aboveground dry weight, and underground dry weight. All data should be measured in triplicate to ensure the reliability and accuracy of the experimental results.

## Results

### Strain MR4

**Fig. 1:**
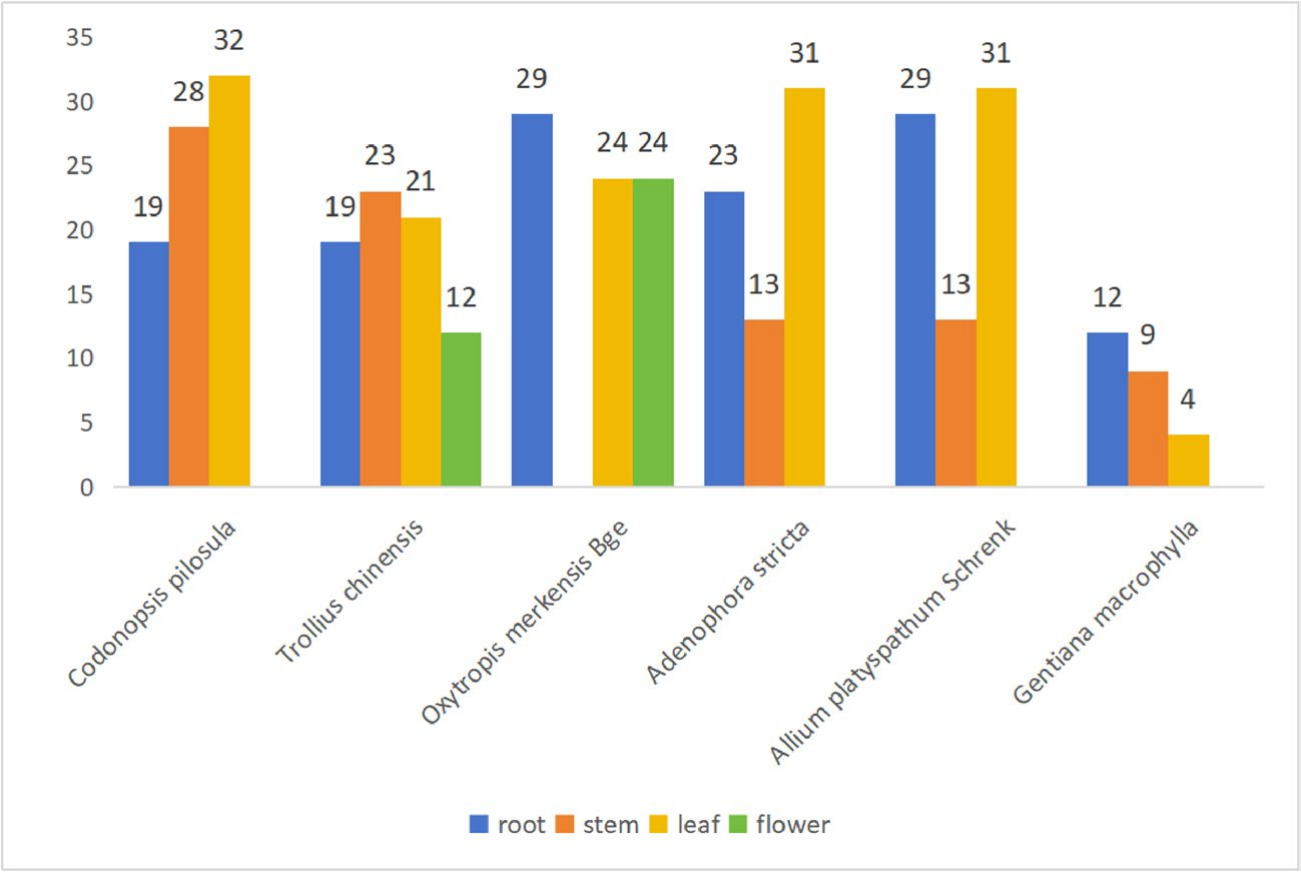
Distribution of endophytic bacteria isolated from different plant tissues

A total of 396 strains of endophytic bacteria were isolated from different tissues of six medicinal plants, including *Allium platyspathum* Schrenk and *Oxytropis merkensis* Bge. The highest number of strains, 79, was isolated from *Allium platyspathum* Schrenk, followed by 77 strains from *Oxytropis merkensis* Bge (Fig. 1). Notably, strain MR4, isolated from *Oxytropis merkensis* Bge, exhibited significant biocontrol potential, inhibiting the cotton fusarium wilt by 60% and cotton verticillium wilt by 48.57%. This strain merits further investigation due to its strong biocontrol capabilities.

### Identification of strain MR4

**Fig. 2:**
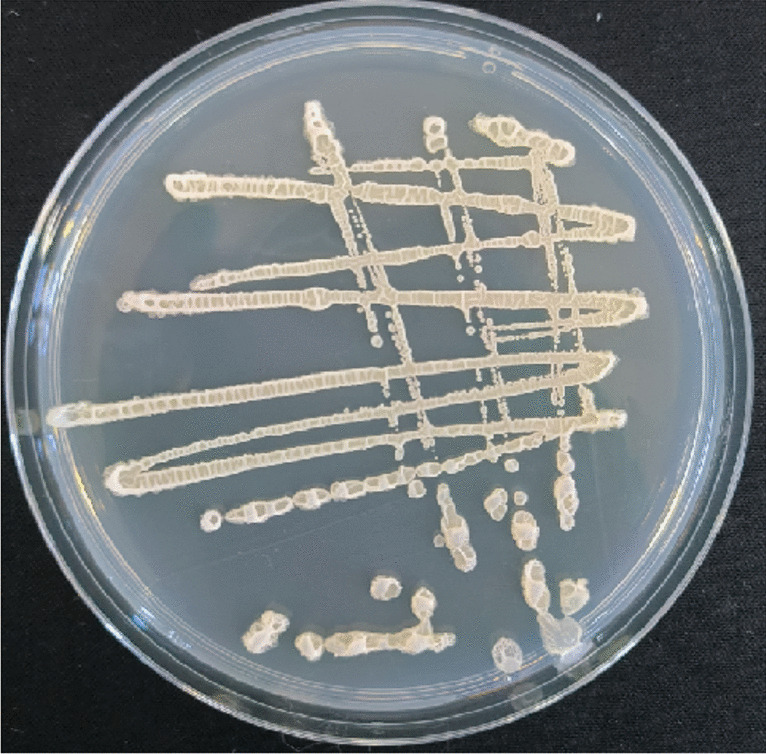
MR4 endophytic bacteria colony diagram

**Fig. 3:**
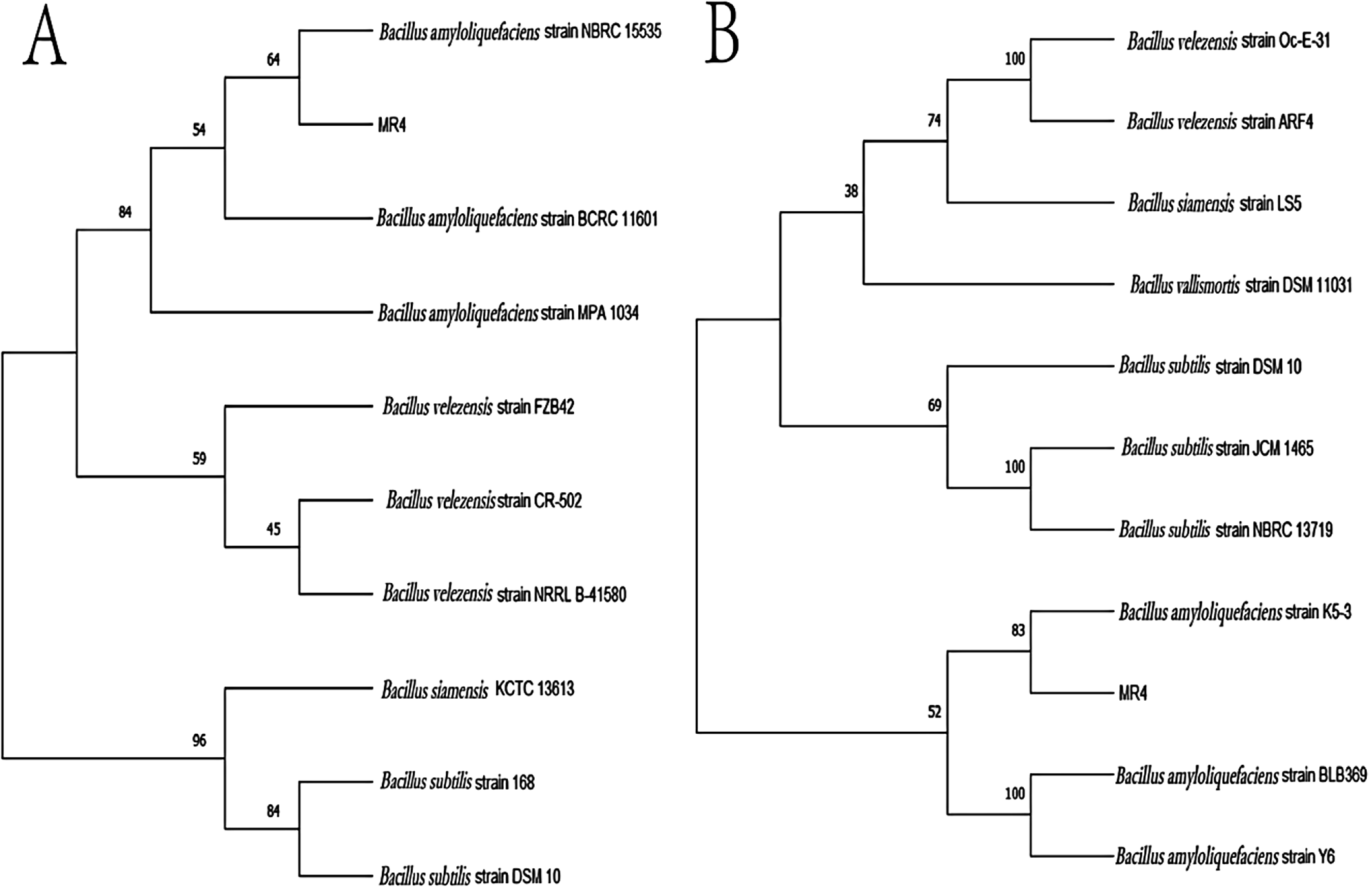
Identification of *Bacillus amyloliquefaciens* MR4 strain. Phylogenetic tree based on nucleotide sequence of 16S rRNA (**A**) and gyrA (**B**)

**Fig. 4:**
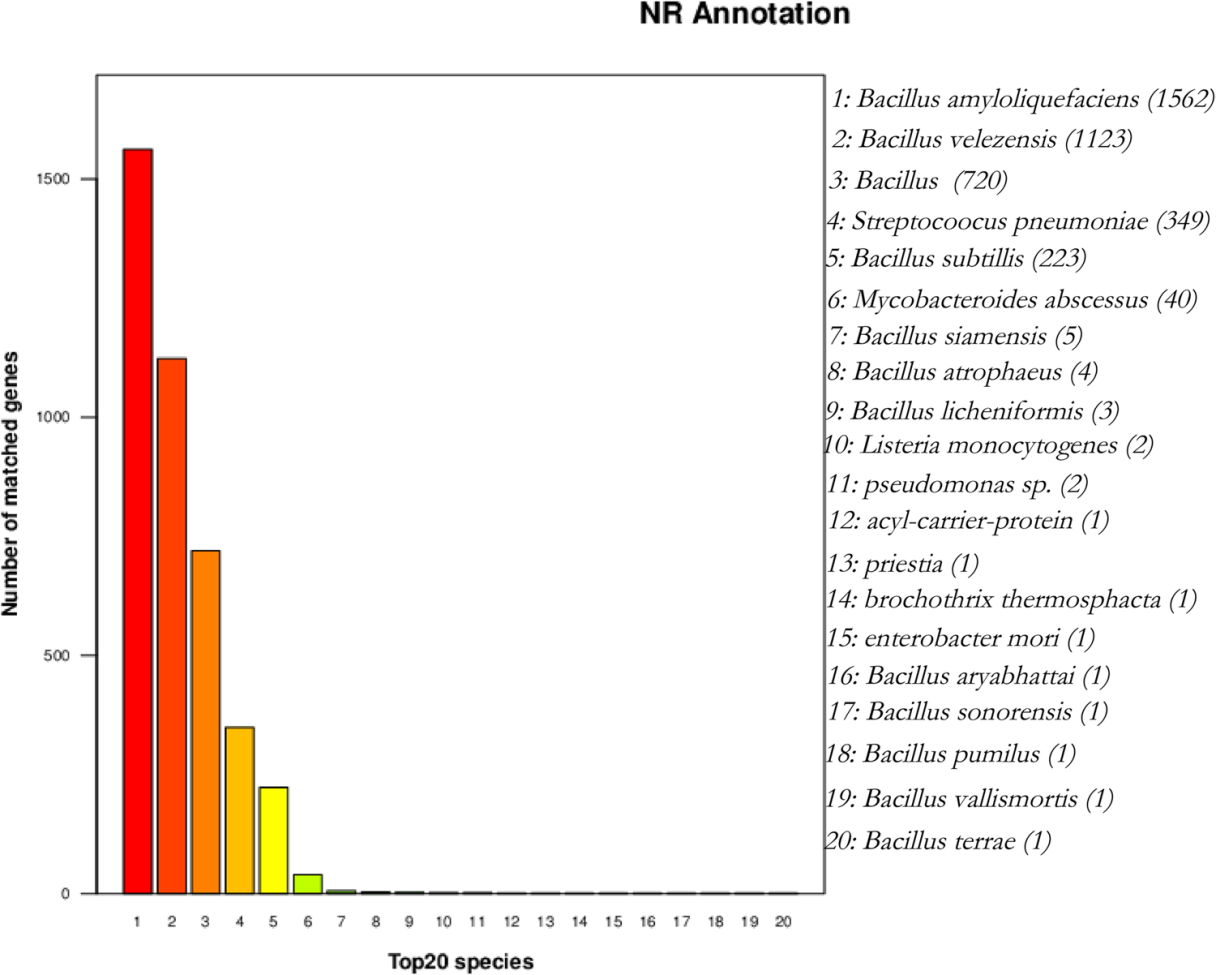
Functional annotation results of NR database of *Bacillus amyloliquefaciens* MR4 genome

**Fig. 5:**
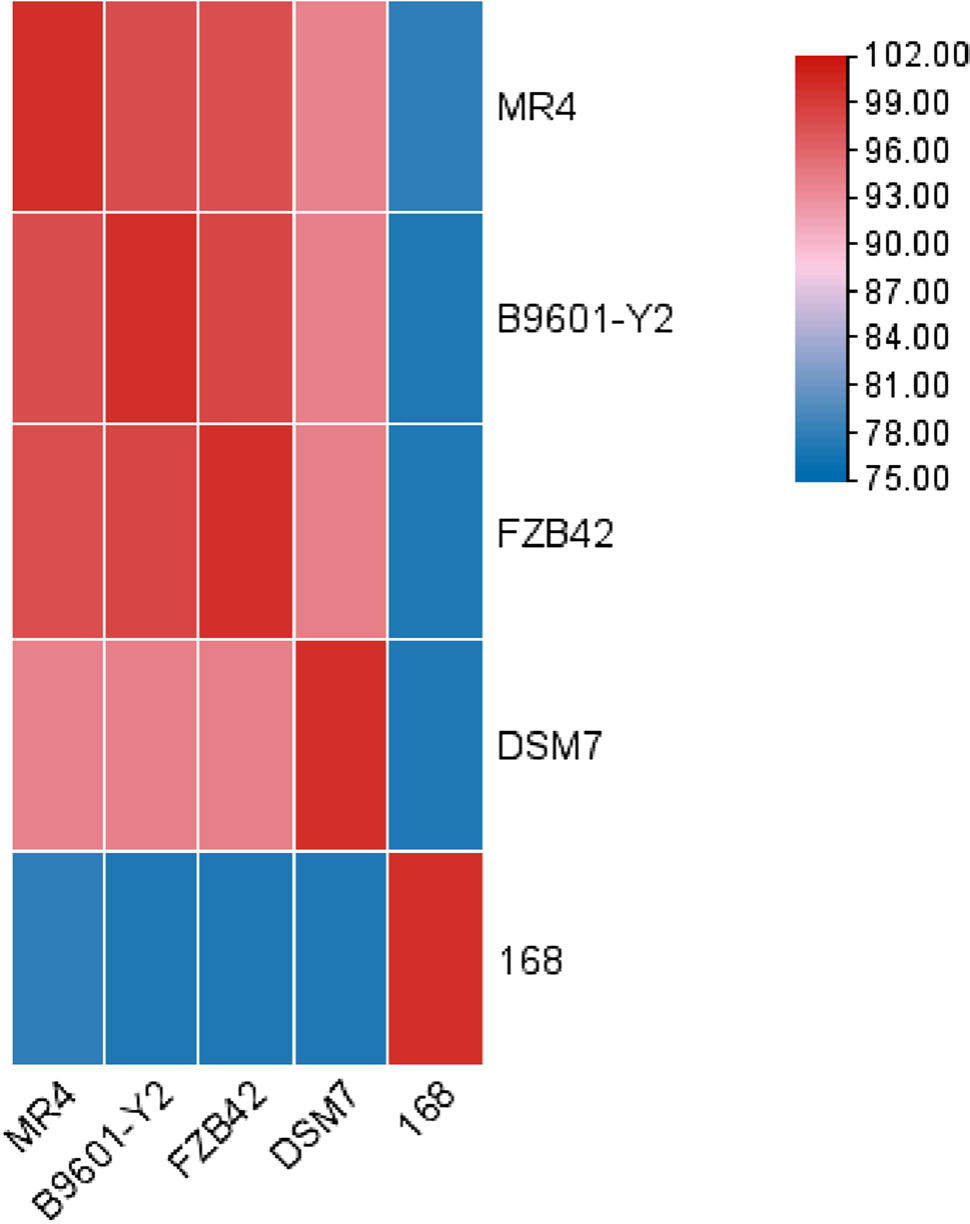
Heatmap of nucleotide consistency between *Bacillus amyloliquefaciens* MR4 and four *Bacillus* strains

When inoculated onto LB medium using the three-zone streaking method and cultured at 37 °C, the colonies of strain MR4 were observed to be opaque and milky white in appearance, with a raised surface and wrinkled edges. When picked, the colonies exhibited a mucous consistency (Fig. 2). Strain MR4 was identified by amplifying partial segments of the 16S rDNA gene and the gyrA gene and then conducting similarity comparison analyses with data in GenBank. Based on the sequences of other standard spore-forming bacteria provided, ten strains with high similarity were selected to construct 16S phylogenetic trees and gyrA phylogenetic trees (Fig. 3A, B). The results indicated that strain MR4 clustered with *Bacillus amyloliquefaciens*, consistent with the annotation results in the NR database (Fig. 4). To further confirm this, nucleotide consistency analysis and heatmaps were generated by comparing MR4 with reference strains *B. amyloliquefaciens* FZB42, *B. amyloliquefaciens* DSM7, *B. amyloliquefaciens* B9601-Y2, and *B. subtilis* 168 (Fig. 5). The results showed that MR4 exhibited a similarity of over 97% with *B. amyloliquefaciens* FZB42 and *B. amyloliquefaciens* DSM7, while its similarity with *B. subtilis* 168 was below 95%, confirming MR4 as *Bacillus amyloliquefaciens*.

### Antagonistic capability of strain MR4 against cotton verticillium and *fusarium* wilts

**Fig. 6:**
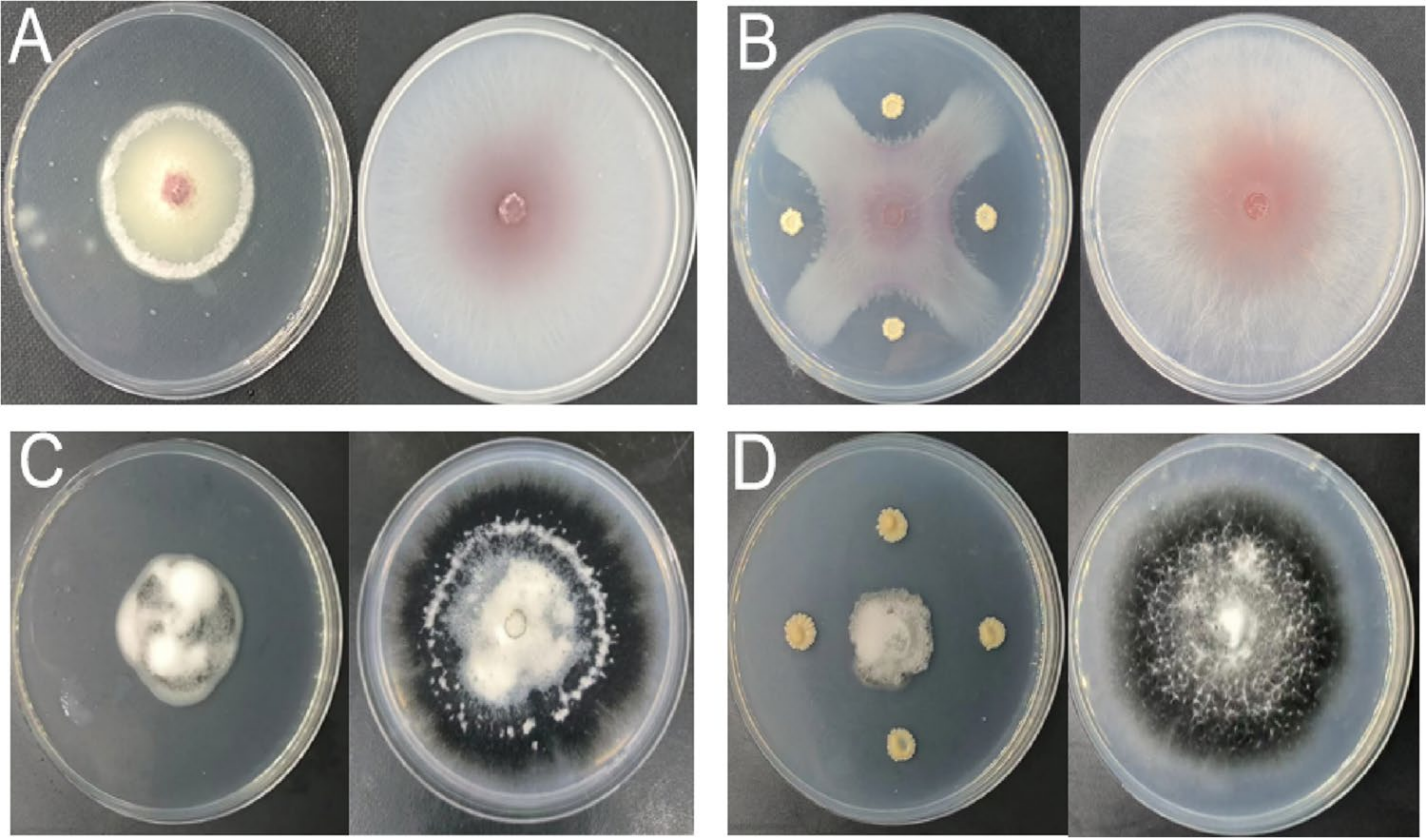
Image of the inhibitory effect of sterile fermentation supernatant (15%) and bacterial suspension on fusarium wilt and verticillium wilt pathogens in cotton. (Note: **A**, **B** the inhibitory effects of MR4 strain supernatant and bacterial suspension on cotton fusarium wilt, respectively. **C**, **D** The inhibitory effects of MR4 non-fermentation supernatant and bacterial suspension on verticillium wilt of cotton, respectively)

In this study, we identified a bacterial strain, MR4, from the rhizosphere of medicinal plants that exhibited significant antagonistic effects against cotton verticillium and fusarium wilts. This finding aims to provide a scientific basis for enhancing disease resistance in cotton crops. In plate co-cultivation experiments, both the supernatant and cell suspension of the MR4 strain demonstrated effective inhibitory effects against cotton verticillium wilt and fusarium wilt (Fig. 6). Specifically, the sterile fermentation supernatant of MR4 inhibited cotton verticillium wilt by 46.54% (Table 2A) and fusarium wilt by 58.09% (Table 2C). Similarly, the MR4 bacterial suspension inhibited cotton verticillium wilt by 58.8% (Table 2B) and fusarium wilt by 72.02% (Table 2D). By quantitatively assessing the inhibitory effects of endophytic bacteria on pathogen growth, this study provides insights for further exploring the potential antagonistic mechanisms of endophytic bacteria and highlights the potential of selecting effective antagonistic bacteria from plant rhizosphere environments as biocontrol agents (Tyśkiewicz et al. 2022).

**Table 2:** Inhibition rate of (A) endophyte aseptic fermentation supernatant on cotton fusarium wilt, (B) endophytic suspensions on cotton fusarium wilt, (C) endophytic supernatant of aseptic fermentation on verticillium wilt of cotton, and (D) endophytic suspensions against verticillium wilt of cotton

Bacterial strain	Colony diameter (cm)		Growth inhibition ratio (%)
MR4	4.31 ± 0.2		46.54
CK	8.07 ± 0.2		0.00
B			
MR4	3.42 ± 0.12		58.8
CK	8.3 ± 0.18		0.00
C			
MR4	3.22 ± 0.09		58.09
CK	7.68 ± 0.19		0.00
D			
MR4	2.15 ± 0.1b		72.02
CK	7.68 ± 0.19		0.00

### Antimicrobial activity genes in MR4

**Fig. 7:**
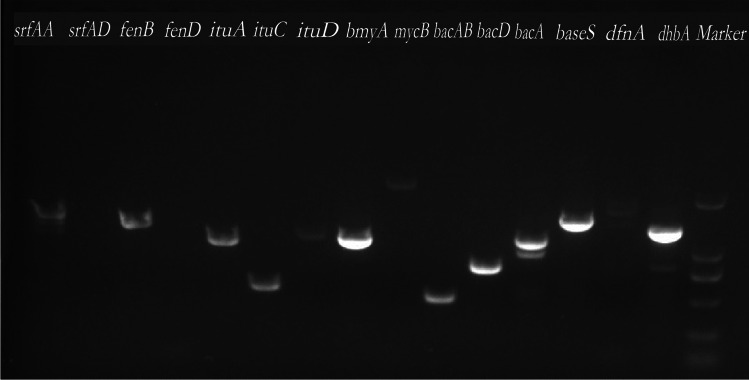
PCR results of MR4 gene encoding antimicrobial substance

Using 15 pairs of primers, PCR amplification of the genes responsible for the synthesis of antimicrobial substances in the antagonistic strain MR4 was performed (Fig. 7). The results showed that MR4 could amplify fragments of the genes srfAA, fenB, ituA, ituC, bmyA, bacAB, bacD, bacA, baseS, dhbA, ituD, and mycB, with approximate sizes of 1600 bp, 1600 bp, 1047 bp, 594 bp, 1200 bp, 500 bp, 815 bp, 1200 bp, 1550 bp, 1350 bp, 1203 bp, and 2000 bp, respectively. These genes encode for seven different non-ribosomal peptides: surfactin, fengycin, iturin, bacillomycin, bacilysin, iturin again, and mycosubtilin. These non-ribosomal peptides exhibit a broad spectrum of antimicrobial activities. For instance, fengycin effectively inhibits fungal diseases in plant root systems, while surfactin demonstrates exceptional performance in promoting plant growth with two polyketide compounds (bacillaene and bacillibactin). Bacillaene inhibits bacterial growth by interfering with the cell division process of pathogenic bacteria (Nakayinga et al. 2021). Bacillibactin, an iron-chelating siderophore, facilitates bacterial iron uptake by binding to iron ions in the environment, thus providing a survival advantage under iron-limited conditions. Although its direct antimicrobial activity may not be pronounced, it plays a crucial role in bacterial growth and survival, indirectly affecting interactions with plants (Luo et al. 2022). The gene for the synthesis of difficidin (dfnA) was not amplified. These results indicate that strain MR4 possesses the capability to synthesize at least eight different types of antimicrobial substances, and it has excellent potential of biocontrol and growth promotion.

### Genome features of MR4

**Fig. 8:**
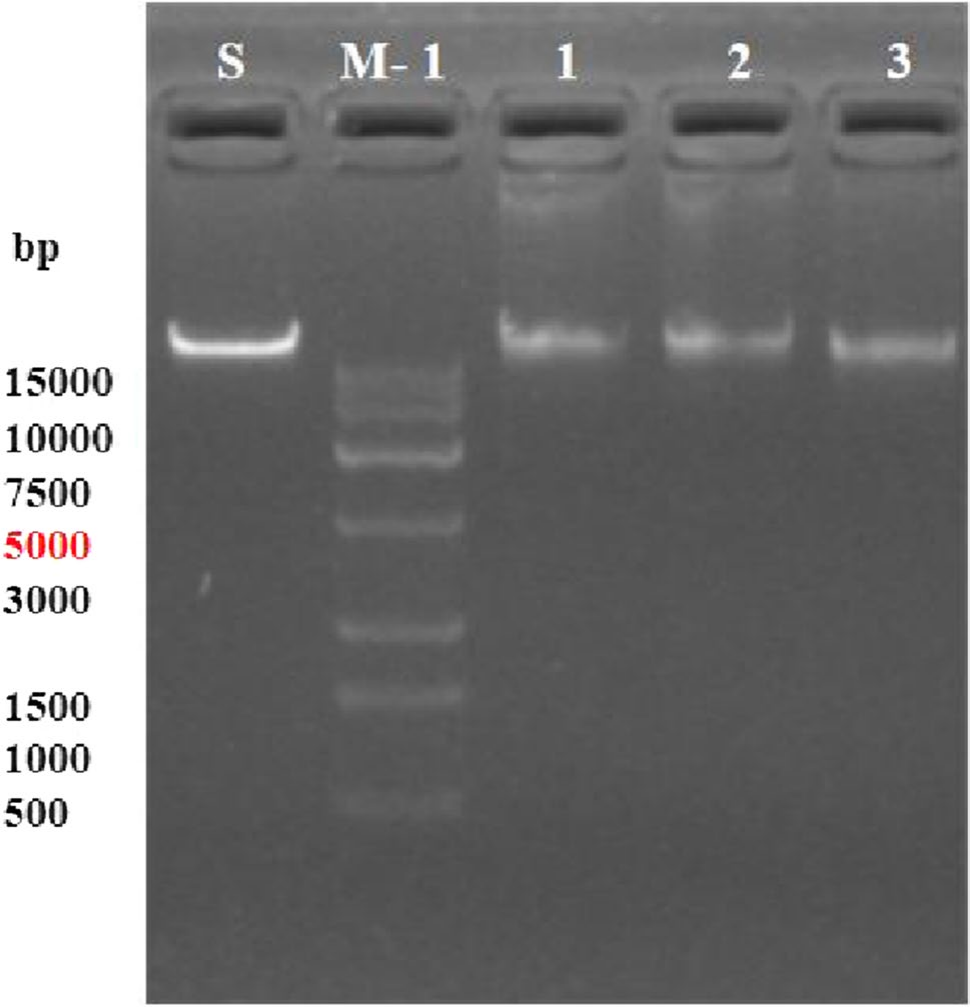
Electrophoretic profile of MR4 (Note: Number 3 refers to MR4)

**Fig. 9:**
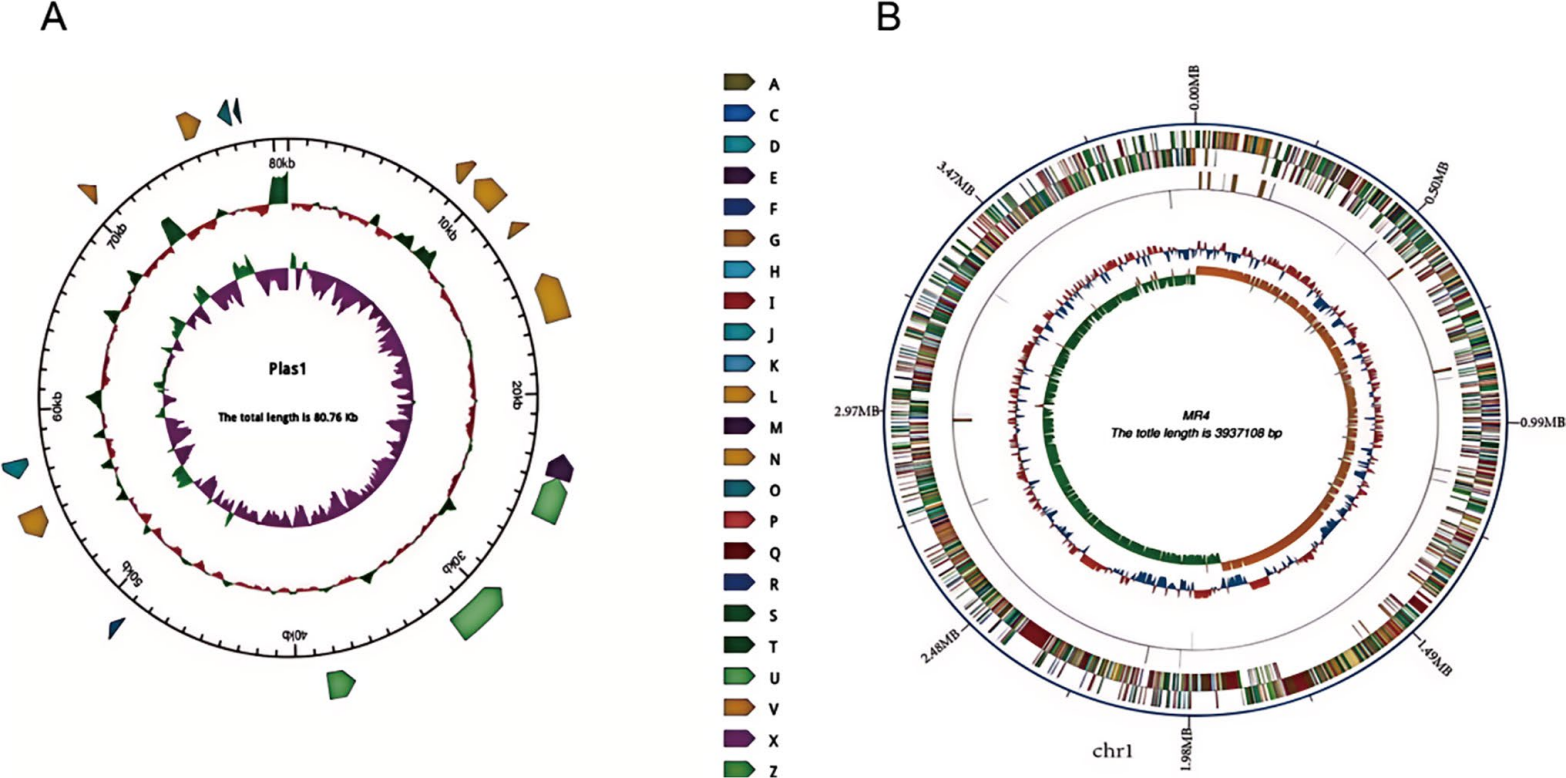
MR4 genome circle map (**A**) and plasmid profile (**B**). (Note: **A** The outermost circle is the position coordinates of the genome sequence. From the outside to the inside, it is the result of gene function annotation, ncRNA, genome GC content, and genome GC skew value. **B** From the outside to the inside, the pictures are COG functional annotation classification genes, genomic sequence position coordinates, genomic GC content, and genomic GC skew value)

The genomic DNA of MR4, extracted and analyzed by agarose gel electrophoresis, displayed a clear band, indicating that the extracted DNA met the quality standards (Fig. 8). Through whole-genome sequencing analysis, we determined the total genome size of the antagonistic strain MR4 to be 4,017,872 base pairs (bp), with a total of 4191 genes encoded. These encoded genes occupy 89.9% of the entire genome. The total length of the encoded genes reaches 3,611,934 bp, with an average gene length of 862 bp and a GC content of 47.14%. The genome consists of a single chromosome and one plasmid. Specifically, the length of the chromosome is 3,937,108 bp with a GC content of 46.58%, while the plasmid length is 80,764 bp with a GC content of 38% (Fig. 9A, B). Within the genome of MR4, we identified 87 tRNA genes, 6 sRNA genes, and 9 each of 5S rRNA, 16S rRNA, and 23S rRNA genes. In addition, eight genomic islands, ten potential prophages, and three CRISPR sequences were discovered. In the gene function annotation process, we identified 3391, 3013, 2748, 3952, and 2748 genes in the Swiss-Prot, COG, GO, KEGG, and Pfam databases, respectively. These annotations provide crucial information about the functional characteristics of the MR4 genome (Table 3). These whole-genome analysis results lay the foundation for further exploration of the biological functions of MR4.

**Table 3:** MR4 annotation results

Locus	Annotation_num
nr	4057
SwissProt	3319
KEGG	3952
COG	3013
TCDB	514
GO	2784
PHI	347
VFDB	193
ARDB	7
CARD	0
Secretory_Protein	88
T3SS	89
CAZy	160
Pfam	2748

### Gene function annotation analysis

#### Basic function annotation analysis

**Fig. 10:**
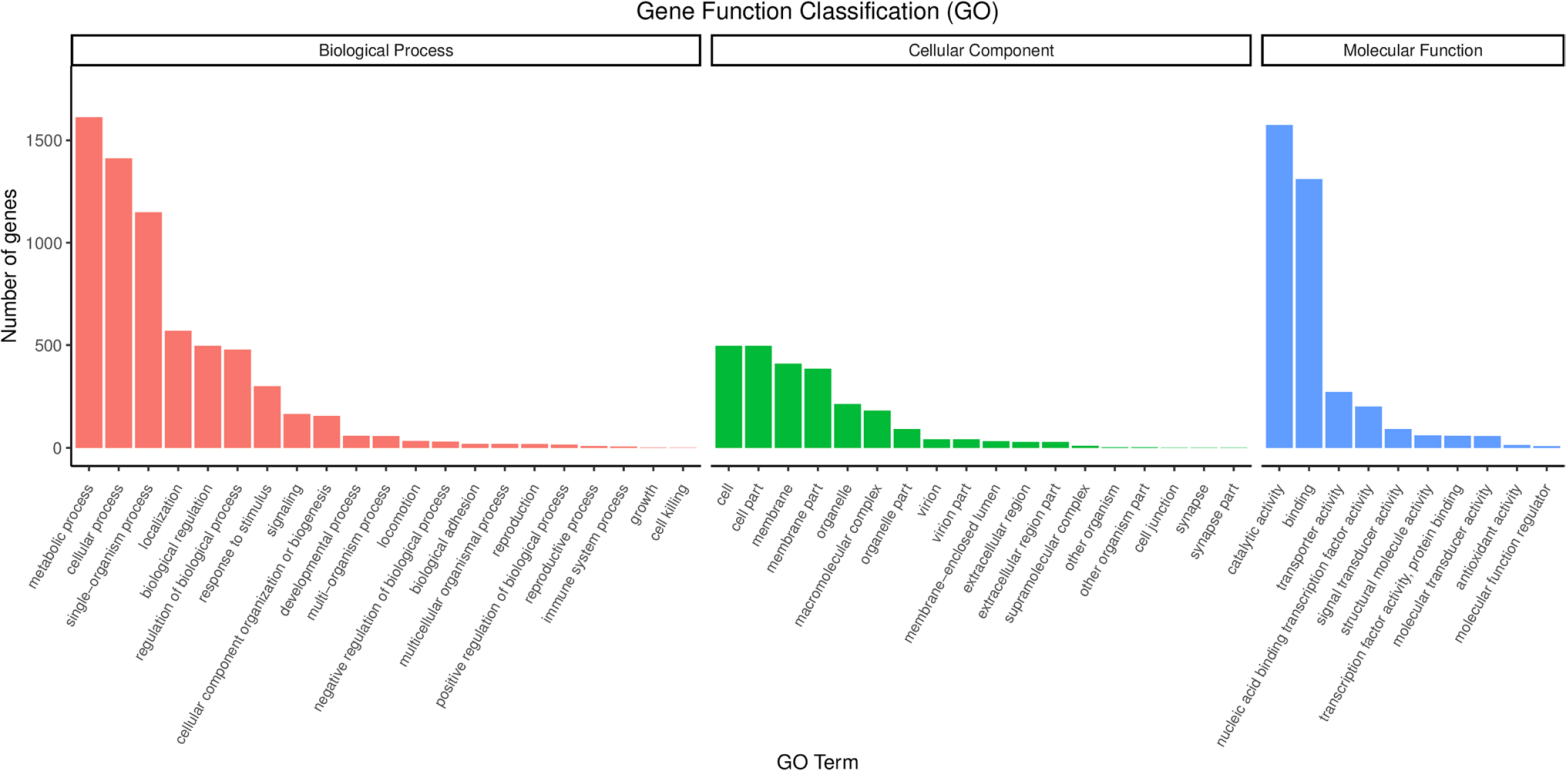
Functional annotation results of GO database of *Bacillus amyloliquefaciens* MR4 genome

**Fig. 11:**
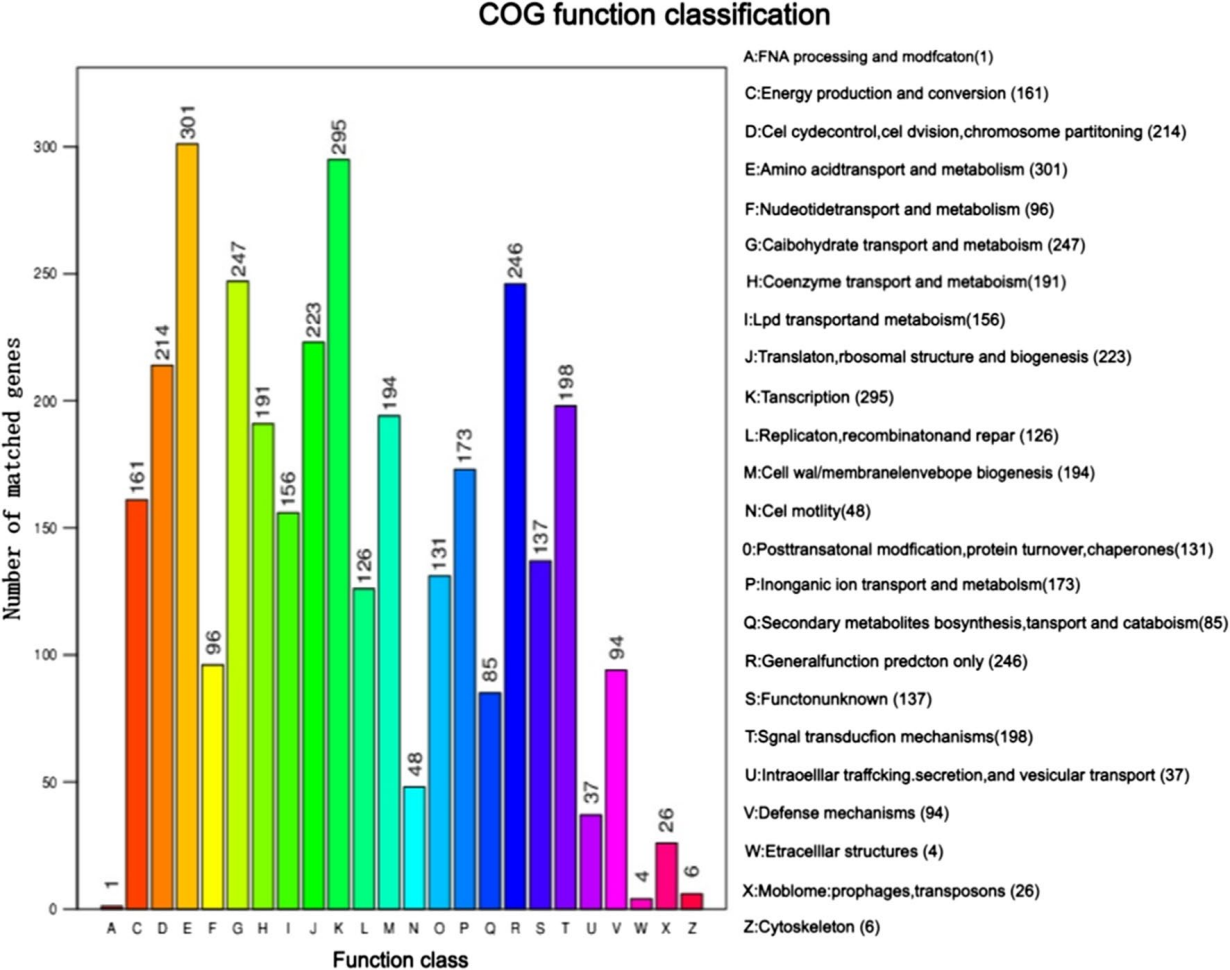
Functional annotation results of COG database of *Bacillus amyloliquefaciens* MR4 genome

**Fig. 12:**
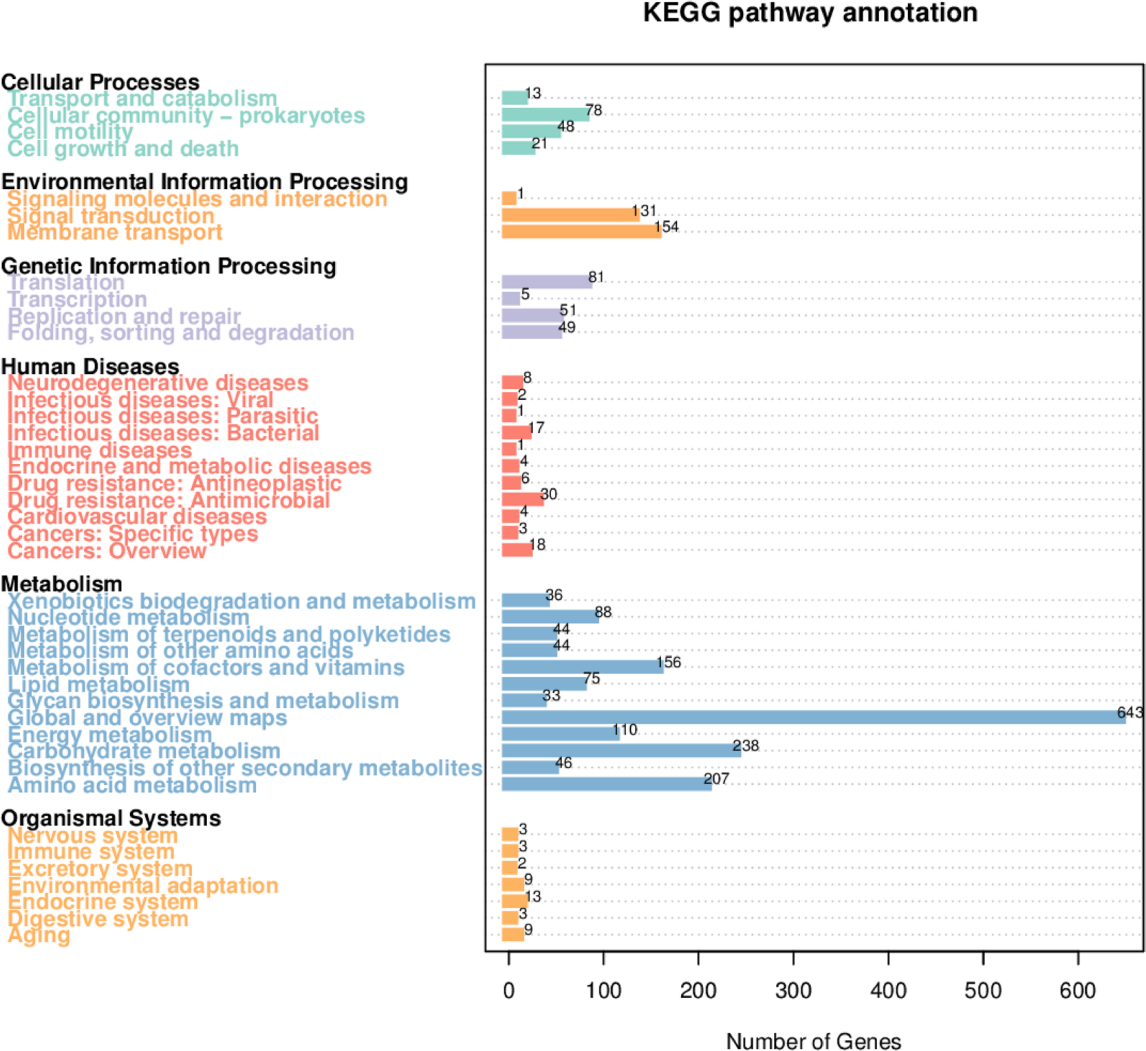
Functional annotation results of KEGG database of *Bacillus amyloliquefaciens* MR4 genome

The GO database is subdivided into three main categories: cellular component, molecular function, and biological process. In the cellular component category, 497 genes are related to cells, with 409 genes associated with the cell membrane. In the molecular function category, the most common annotations are catalytic activity and binding functions, linked to 1575 and 1311 genes, respectively. In the biological process category, the highest number of genes is related to metabolism, totaling 1613 genes (Fig. 10). This suggests that the MR4 strain may possess strong metabolic capabilities and environmental adaptability, which could be crucial for its survival in the environment and interactions with hosts. Protein annotation of MR4 was performed using the COG database. The classification of the 3390 genes annotated by COG for strain MR4 is shown in Fig. 11. Among them, there are 1015 genes related to amino acid transport and metabolism. Additionally, the most abundant annotation is for transcription, with 295 genes, accounting for 8.7% of the annotated genes. This is followed by genes related to carbohydrate transport and metabolism, as well as general function prediction, with 247 and 246 genes, respectively (Fig. 11). In the KEGG database, the MR4 genome has been annotated with 3764 genes, covering six major categories, including cellular processes, environmental information processing, genetic information processing, human diseases, metabolism, and biological systems. Particularly noteworthy are the metabolic pathways and environmental information processing pathways, encompassing 1720 and 286 genes (Fig. 12). Studying these pathways and genes can provide deeper insights into the microbial ability to degrade harmful substances and adapt to the environment. Interpreting this information will provide important clues for understanding the biological characteristics and potential applications of microorganisms. In the Pfam (Protein families) database, the most common functional domain is the P-loop containing the NTPase superfamily, with 1220 genes containing this structural domain. The CAZy (Carbohydrate-Active enZYmes) database identified 166 genes. The Swiss-Prot database indicates the presence of three ATP-binding proteins LnrL (Linearmycin Resistance ATP-binding Protein LnrL) with linearmycin resistance activity, as well as three polyketide synthases PksM (Polyketide Synthase PksM). The ATP-binding protein LnrL conferring linearmycin resistance is an ABC transporter protein, primarily aiding bacterial cells in excluding harmful substances and speculated to play a crucial role in biocontrol. However, the specific mechanism of action of LnrL protein in cell signaling and disease progression remains unclear and requires further investigation (Zhang et al. 2023). Polyketide synthase PksM is involved in the synthesis of polyketide compounds, which often possess significant biological activities, including antibiotic effects. The comprehensive annotation results from multiple databases indicate that *Bacillus amyloliquefaciens* has strong biocontrol potential and merits further exploration.

#### Prediction of MR4 secondary metabolite gene clusters

**Fig. 13:**
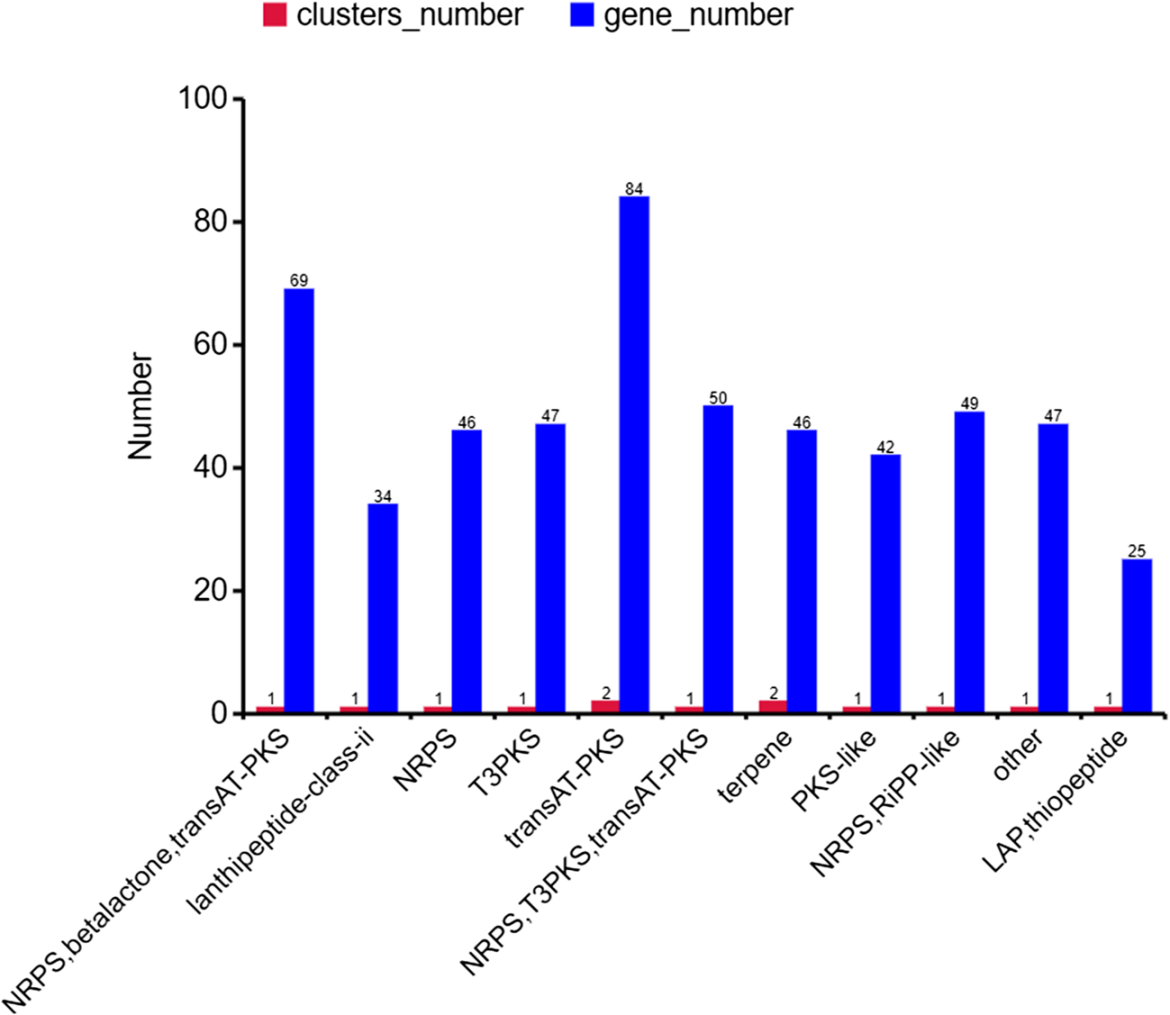
Annotation results of the secondary metabolite gene clusters in *Bacillus amyloliquefaciens* MR4

We utilized the antiSMASH database for prediction and identified a total of 13 secondary metabolite biosynthetic gene clusters in the MR4 genome (Table 4, Fig. 13). These clusters include NRPS, betalactone, transAT-PKS, lanthipeptide-class-ii, NRPS, T3PKS, NRPS, T3PKS, transAT-PKS, PKS-like, NRPS, RiPP-like other, LAPthiopeptide, terpene, and transAT-PKS. Among them, trans-PKS and terpene are dominant gene clusters, each composed of two gene clusters. The transAT-PKS gene cluster may play a role in the production of specific polyketide secondary metabolites, such as antibiotics, pigments, or toxins. The identified two terpene gene clusters are crucial components for the biosynthesis of terpene compounds in plants, playing a significant role in plant biosynthesis and the interaction between plants and the environment (Chu et al. 2011). We found that the majority of the predicted gene clusters are associated with antifungal effects.

**Table 4:** MR4 secondary metabolic gene cluster

Gene cluster	From	To	Gene cluster type	Similarity (%)	Similar gene cluster
Cluster 1	307,259	372,096	NRPS	82.00%	Surfactin
Cluster 2	580,479	609,648	Thiopeptide, LAP	4.00%	Ijanimicin
Cluster 3	920,097	961,341	PKS-like	7.00%	Butirosin A, butirosin B
Cluster 4	1,046,247	1,063,543	Terpene	−	−
Cluster 5	1,183,698	1,212,587	Lanthipeptide-class-ii	−	−
Cluster 6	1,376,220	1,462,648	transAT-PKS	100.00%	Macrolactin
Cluster 7	1,688,546	1,788,885	transAT-PKS, T3PKS,NRPS	100.00%	Bacillaene
Cluster 8	1,854,640	1,990,780	NRPS, transAT-PKS, betalactone	100.00%	Fengycin
Cluster 9	2,014,378	2,036,261	Terpene	−	−
Cluster 10	2,107,025	2,148,131	T3PKS	−	−
Cluster 11	2,318,344	2,412,120	transAT-PKS	100.00%	Difficidin
Cluster 12	3,028,372	3,080,160	NRPS, RiPP-like	100.00%	Bacillibactin
Cluster 13	3,598,185	3,639,603	Other	100.00%	Bacilysin

#### Antagonistic potential

**Fig. 14:**
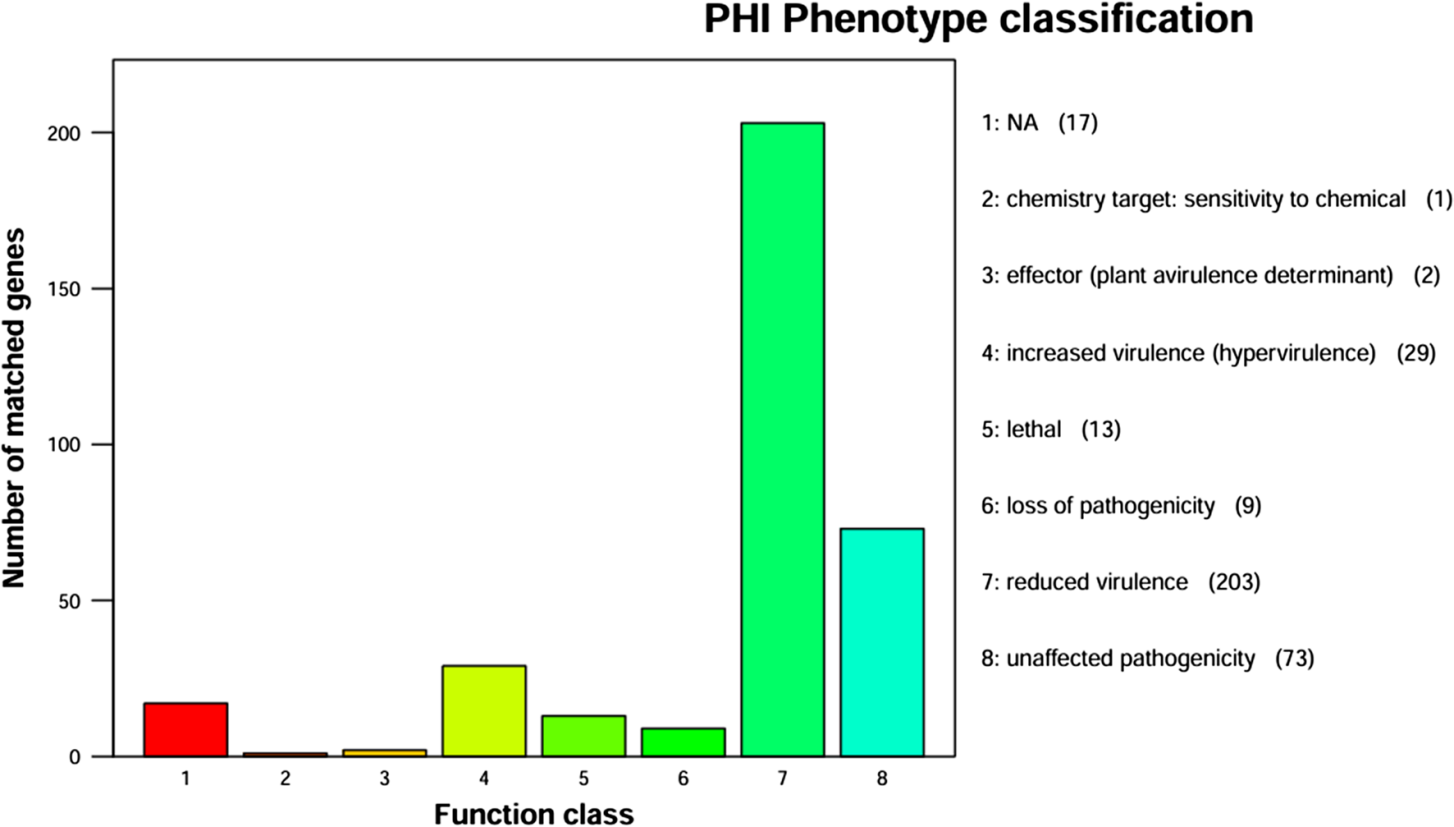
Functional annotation results of PHI database of *Bacillus amyloliquefaciens* MR4 genome

The Pathogen-Host Interaction Database (PHI) is an important biological information resource with significant implications for studying potential target genes in drug intervention. The PHI database records various gene variations related to pathogen traits. The annotation results of MR4 in the PHI database (Fig. 14) indicate that gene mutations that weaken pathogen virulence are the most common, with a total of 203 mutations. Next are gene mutations that do not affect pathogenicity, totaling 73. Additionally, there are relatively more gene mutations that enhance pathogen virulence, reaching 29. Gene mutations that completely lose pathogenicity amount to 9, while lethal gene mutations total 13. These data not only reveal the number of mutated genes but also indicate their specific locations in the genome, providing a solid theoretical basis and direction for further in-depth studies of this strain. The Virulence Factor Database (VFDB) focuses on studying the pathogenic factors of pathogenic bacteria, chlamydia, and other pathogenic microorganisms. It provides species information on virulence genes, basic characteristics descriptions, and detailed explanations of virulence gene functions and pathogenic mechanisms. Through VFDB annotation analysis, 198 gene annotations were obtained, mainly involving bacterial adhesion, secretion system proteins, and transcription factors. In the results from the Antibiotic Resistance Genes Database (ARDB), annotations for seven types of resistance genes were identified, including lincomycin, phosphomycin, tetracycline, and others. It is speculated that *Bacillus amyloliquefaciens* may express resistance to these antibiotics and pump them out through efflux resistance proteins to protect itself and develop resistance.

### Comparison of MR4 with *Bacillus* spp. strains

**Fig. 15:**
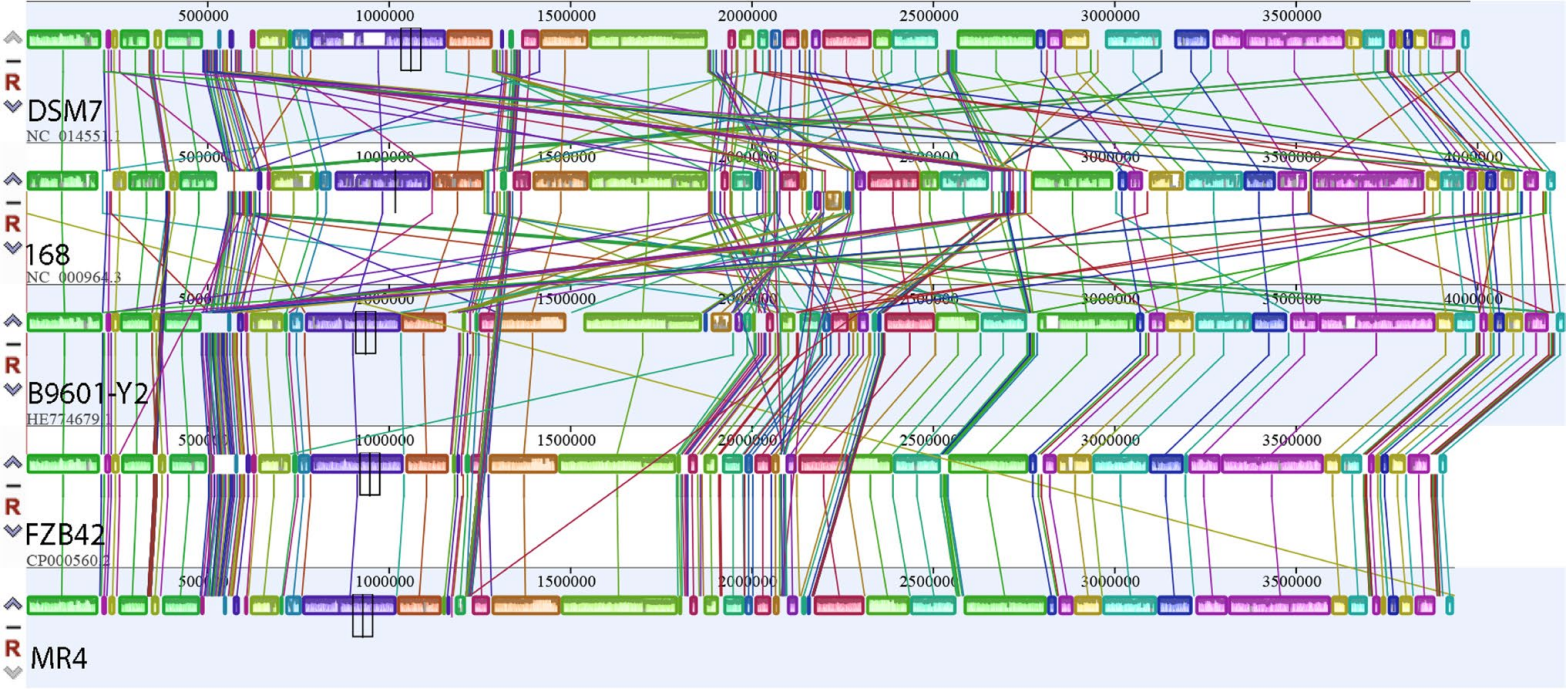
Collinearity relationship diagram between *Bacillus amyloliquefaciens* MR4 and four model strains

We found that the size of the MR4 genome is similar to that of four *Bacillus* strains, with the closest match being to *B. amyloliquefaciens* DSM7. The GC content is most similar to that of *B. amyloliquefaciens* FZB42, followed by *B. amyloliquefaciens* DSM7. The number of tRNAs is comparable to that of *B. subtilis* 168 and lower than the other three strains of starch-degrading *Bacillus* (Table 5). The genome of this strain exhibits widespread evolutionary events such as gene inversion and gene recombination. By comparing homologous regions in the genome sequences using alignment tools, evolutionary processes can be determined. To assess the evolutionary distance between the five strains, their whole-genome sequences were compared using the MAUVE program with default parameters (Fig. 15). The alignment results show that there are significant gene insertions or deletions and locally collinear blocks (LCBs) inversions between DSM7, 168, and B9601-Y2. The genomes of strains MR4 and FZB42 exhibit good collinearity, with no extensive rearrangements or deletions, but transpositions and insertions are still present. Overall, strain MR4 shows a closer genetic relationship with *B. amyloliquefaciens* FZB42 among the five strains of *Bacillus*.

**Table 5:** Basic characteristics of the whole-genome sequences of MR4 and four related strains

Items	*B. amyloliquefaciens* FZB42	*B. subtilis* 168	*B. amyloliquefaciens* DSM7^T^	*B. amyloliquefaciens* B9601-Y2	MR4
Genome size (bp)	3,918,589	4,214,630	3,980,199	4,238,624	4,017,834
GC content (%)	46.4	43.5	46.1	45.8	47.14
Protein-coding sequences	3693	4106	3982	4159	4191
Number of tRNAs	89	86	93	91	87
GenBank sequence	CP000560	NC_000964.3	NC_014551.1	HE774679	CP146236

### Analysis of the biocontrol and growth-promoting potential of MR4

**Fig. 16:**
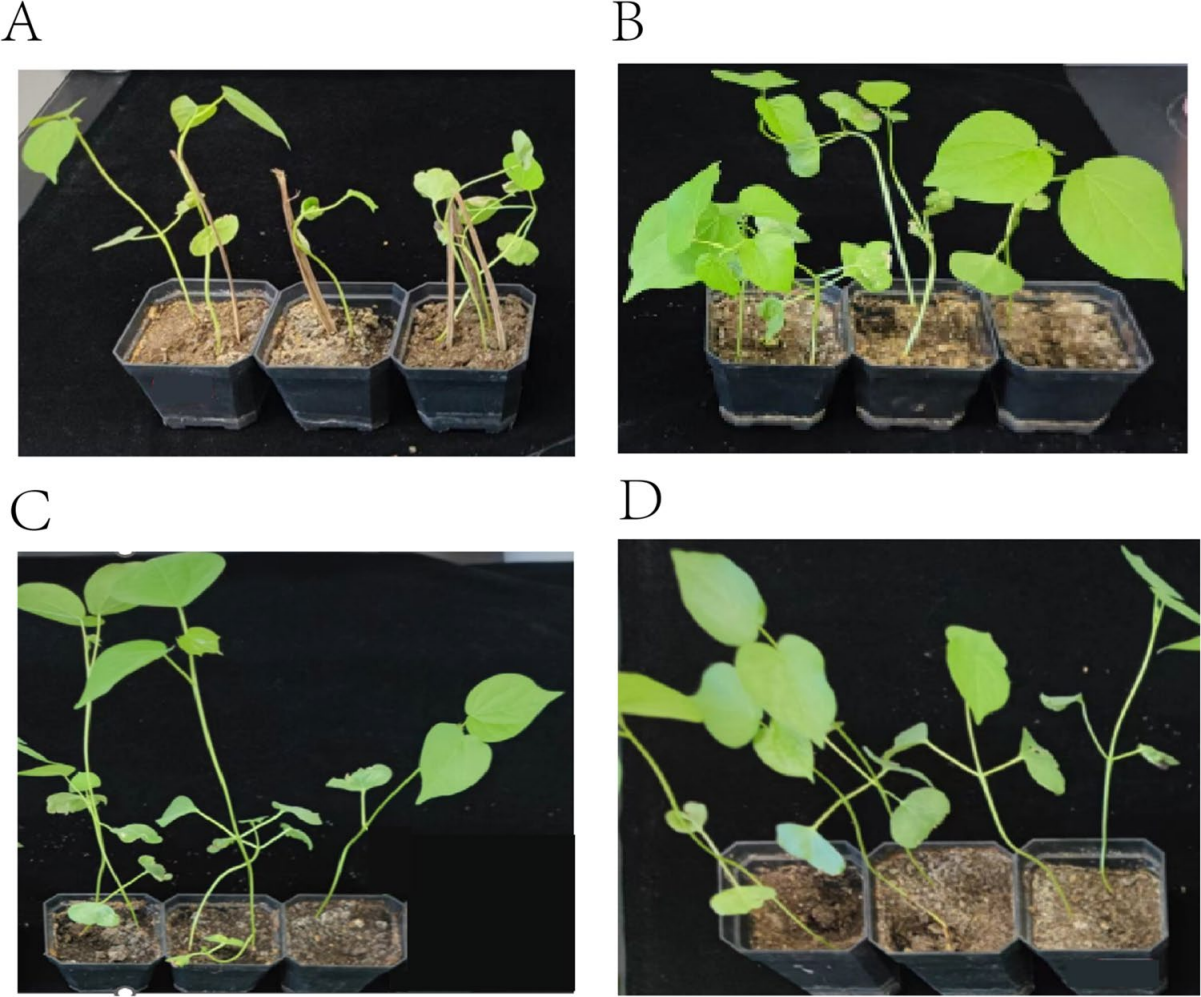
**A** Xin Hai 21 control; **B** Xin Hai 21 treated with MR4; **C** Xin Lu Zhong 63 control; **D** Xin Lu Zhong 63 treated with MR4

Through the pot culture experiment, we observed that the two cotton varieties, Xin Hai 21 and Xin Lu Zhong 63, demonstrated better growth and healthier conditions after inoculation with the MR4 strain compared to the untreated control group. Specifically, the cotton plants inoculated with MR4 had broader and fuller leaves with a more vibrant green color, whereas the untreated control group exhibited slight leaf chlorosis and narrower leaves (as shown in Fig. 16).

According to the agronomic trait measurements, the average plant height of Xin Hai 21 cotton inoculated with MR4 was 32.4 cm, the average fresh weight was 1.8 g, the average dry weight was 0.2 g, and the average leaf count was 5 leaves. In contrast, the corresponding data for the Xin Hai 21 control group were 25.7 cm, 1.6 g, 0.17 g, and 4 leaves, respectively. For Xin Lu Zhong 63 cotton, the average plant height after MR4 inoculation was 31.0 cm, the average fresh weight was 1.6 g, the average dry weight was 0.18 g, and the leaf count was 5 leaves. The corresponding data for the control group were 28.3 cm, 1.5 g, 0.17 g, and 5 leaves (detailed in Supplementary Tables 1 and 2).

Overall, the cotton plants inoculated with MR4 showed superior performance in terms of average plant height, fresh weight, dry weight, and leaf count compared to the control group. This indicates that the MR4 strain has the potential to promote cotton growth and enhance plant health. These results support the potential application of the MR4 strain as a biocontrol agent and growth promoter.

## Discussion

Biological control not only offers an environmentally friendly and sustainable method for managing crop diseases but also reduces dependence on chemical pesticides, thereby lowering the risk of chemical residues in agricultural production and their negative impact on ecosystems (Liu and Yang 2022). Biological control can enhance crop resistance to diseases and promote the health and balance of agricultural ecosystems (Zhang et al. 2023). This approach not only helps protect consumer and crop safety but also supports the maintenance of biodiversity and the promotion of sustainable agricultural development (Liu and Yang 2022). Therefore, biological control is one of the key strategies for achieving environmentally friendly and efficient production goals in modern agriculture. The MR4 strain identified in this study, with its unique capabilities in biological control and cold resistance, stands out among other endophytic bacteria. In the field of agricultural biological control, finding microbial resources that can effectively combat pathogens and adapt to extreme environmental conditions is of great significance. The discovery of the MR4 strain not only enriches the agricultural microbial resource bank but also provides new solutions for controlling plant diseases under extreme conditions such as low temperatures.

*Bacillus* species are commonly used biosynthetic bacteria that play a significant role in biological control through various mechanisms such as direct antagonism against pathogens, activation of the plant’s innate defense mechanisms, promotion of beneficial microbial growth, and improvement of soil health (Luo et al. 2022). In this study, the plate antagonism test showed that *Bacillus amyloliquefaciens* strain MR4 exhibited strong inhibitory effects against cotton verticillium wilt. Previous studies have also demonstrated the excellent disease control capabilities of *Bacillus amyloliquefaciens* against diseases such as rice bacterial blight, tomato early blight, and cucumber downy mildew (Wei and Shuo 2021). Combining the results of antimicrobial gene PCR amplification and the prediction of secondary metabolite biosynthetic gene clusters from the antiSMASH database, we found that both contain various lipopeptides, including surfactins, iturins, and fengycins. These lipopeptides integrate into the cell membranes of fungi and bacteria, altering their permeability, leading to the leakage of cell contents, and ultimately causing cell death. Iturins and fengycins, in particular, show high efficacy against fungal pathogens by disrupting the integrity of cell membranes through interaction with specific components, such as sterols (Yan et al. 2023). Bacillibactin, detected in this study, is an iron siderophore that binds to environmental iron ions, aiding bacterial iron absorption and providing a survival advantage under iron-limited conditions. Iron is a central component of various plant enzymes, involved in processes such as rhizosphere colonization, induction of systemic resistance, and more. Effective iron management strategies are crucial for promoting plant health and disease management in practice. This includes rational fertilization plans to ensure sufficient iron supply to plants, the use of iron chelators to enhance iron availability, and the utilization of siderophore-producing microbes to improve the bioavailability of iron and enhance biological control effects (Fei et al. 2018). The absence of difficidin (dfnA) in PCR amplification results, a compound known for its broad-spectrum antibiotic activity against many Gram-positive and Gram-negative bacteria, warrants further investigation into the reasons for its absence. MR4’s ability to synthesize a variety of different antimicrobial substances demonstrates its broad inhibitory potential against plant pathogens. The diversity of these antimicrobial substances not only illustrates MR4’s adaptability and competitive survival but also lays the foundation for the development of new biopesticides. Further research and development of these antimicrobial substances could lead to safer and more effective biological control strategies.

Overall, this study provides a theoretical groundwork for the agronomic utilization of MR4, presenting it as a viable microbial resource for biocontrol and plant growth enhancement. The findings contribute to the pool of sustainable agricultural practices, addressing the limitations of chemical pesticides and paving the way for more eco-friendly and effective disease management strategies in crop production. Further research should focus on the following areas: (1) In-depth study of antimicrobial mechanisms: Although this study revealed MR4’s antimicrobial potential through genomic and metabolite analysis, its specific mechanisms of action require further investigation. Particularly, the synergistic effects of multiple gene clusters and the impact of environmental factors on antimicrobial efficacy need more experimental validation. (2) Optimization of application conditions: The effectiveness of MR4 in real-world agricultural applications may be influenced by factors such as soil type, climate conditions, and crop species. Therefore, additional field trials and condition optimization will help improve the practical application of MR4. (3) Development of biocontrol agents: Based on MR4’s antimicrobial properties, new biocontrol agents can be developed. These agents need to demonstrate their efficacy not only under laboratory conditions but also in field conditions to verify their feasibility and stability. (4) Long-term ecological impact assessment: While biological control is considered environmentally friendly, the long-term ecological impacts of any new biocontrol agent must be assessed to ensure its safety for non-target organisms and overall ecosystems. Through these further studies and developments, the MR4 strain is expected to play a more significant role in modern agriculture, providing a sustainable and efficient method for crop disease management.

## Supplementary Information

Below is the link to the electronic supplementary material.

Supplementary file1 (XLSX 12 KB)

Supplementary file2 (DOCX 16 KB)

## Data Availability

The pure culture of *B. amyloliquefaciens* MR4, sequenced in this study, is preserved in the Key Laboratory of Integrated Pest Management (IPM) of Xinjiang Production and Construction Corps in Southern Xinjiang, College of Agronomy, Tarim University, China. The whole genomic data obtained in this study are available both at NCBI GenBank under BioProject accession number PRJNA1070814 and BioSample ID SAMN39661546. The genome accession number of *B. amyloliquefaciens* MR4 is CP146236 (Chr1) and CP146237 (plas1).
