# Supplementary material for: Comparative genome analysis of endophytic Bacillus amyloliquefaciens MR4: a potential biocontrol agent isolated from wild medicinal plant root tissue
Source: J Appl Genet. 2024 Sep 30;65(4):907–23. doi: 10.1007/s13353-024-00905-9 (PMC11561014; doi:10.1007/s13353-024-00905-9)
Supplement: Supplementary file 2 — Supplementary file2 (DOCX 16 KB) [file 13353_2024_905_MOESM2_ESM.docx]

**Isolation and Screening of Antagonistic Strains**

Tissue separation method was used to isolate and purify endophytic bacteria from different tissues of medicinal plants. Roots and stems were cut into small segments of about 3-5 cm, and leaves were cut into squares of about 3×3 cm^2^. The following disinfection procedure was applied: soaking in 75% (v/v) ethanol solution for 30 s, rinsing 2-3 times with sterile water, soaking in 3% (w/v) NaClO solution for 3 min, and finally rinsing 2-3 times with sterile water. The surface-sterilized root and stem tissues were cut into small segments of about 0.5 cm, and the leaves were cut into tissue blocks of 0.5×0.5 cm^2^. Using a sterilized scalpel, roots and stems were bisected, and the cut surface was placed on the culture medium, incubated at 30°C for 24 h. The sterile water from the last washing was spread on the culture medium and incubated for 24 h to verify complete disinfection. Pure colonies were inoculated into tubes containing 5 mL of liquid LB medium, and incubated overnight at 30°C, 180 rpm in a constant temperature shaking incubator. Using the plate confrontation method, a 5 mm Pathogen block. was introduced into the center of the medium, and 2 μL of the prepared endophytic bacterial liquid was introduced 25 mm from the Pathogen block., with liquid LB introduced in the control group in the same amount. Each treatment was repeated 3 times. When the control group’s pathogen covered the culture medium, the mycelial growth diameter was measured and the inhibition rate was calculated as (control pathogen diameter - test pathogen diameter) / control pathogen diameter × 100%.

**Determination of the Inhibition Rate of Strain MR4 against Cotton Verticillium Wilt**

Co-culture method to determine the effect of sterile fermentation broth of Bacillus on the growth of cotton wilt pathogen colonies. The antagonist bacteria were inoculated into LB liquid medium and cultured at 30°C, 180 r/min for 48 h. 40 mL of antagonist bacterial liquid was placed in a 50 mL centrifuge tube and centrifuged at 6000 r/min $for 10 min. In a sterile operation bench, the supernatant was filtered through a microporous membrane (0.22 μm) with a sterile syringe to obtain sterile fermentation broth. 100 μL of sterile fermentation broth was spread on an LB plate and incubated at 30°C for 24 h to ensure the supernatant was sterile. With a 15% (V/V) addition, the supernatant of the fermentation broth was mixed with PDA, and 15 mL of sterile fermentation broth was added to 100 mL of melted PDA medium at about 55°C, mixed well, and poured into a plate (9 cm). After the plate solidified completely, a 5 mm diameter Pathogen block. that had been activated was inoculated into the center of the PDA plate. Each treatment was repeated 3 times, and an equal volume of LB liquid medium was added as a control. The plates were incubated at a constant temperature of 28°C. After 7 days, the pathogen colony diameter was measured using a cross method, and the inhibition rate was calculated using Excel, with statistical analysis performed using SPSS. The inhibition rate (%) = (control pathogen colony diameter - treatment pathogen colony diameter) / control pathogen colony diameter × 100%.

Confrontation culture method to determine the effect of Bacillus suspension on the growth of cotton wilt pathogen colonies. The bacterial precipitate after centrifugation was washed 2-3 times with sterile water and mixed with sterile water, adjusting the OD600=1 to obtain the antagonistic bacterial suspension. A 5 mm Pathogen block. was inoculated into the center of a 9 cm diameter PDA plate. At four points perpendicular to the Pathogen block., 25 mm away, 2 μL of the antagonistic bacterial suspension was added, left for 10-20 min, and then incubated in a constant temperature incubator at 27°C. Sterile water was used as a control to observe the antagonistic situation and record and measure the colony diameter.

The co-culture method to determine the effect of sterile fermentation broth of Bacillus on the growth of cotton Fusarium wilt pathogen colonies was the same as method (1), with the pathogen being cotton Fusarium wilt pathogen (V. dahliae).

The confrontation culture method to determine the effect of Bacillus suspension on the growth of cotton Fusarium wilt pathogen colonies was the same as method (2), with the pathogen being cotton Fusarium wilt pathogen (V. dahliae).
